# Cortisol modulates calcium release-activated calcium channel gating in fish hepatocytes

**DOI:** 10.1038/s41598-021-88957-3

**Published:** 2021-05-05

**Authors:** Chinmayee Das, Manoj K. Rout, Willem C. Wildering, Mathilakath M. Vijayan

**Affiliations:** 1 Department of Biological Sciences, University of Calgary, 2500 University Drive NW, Calgary, AB T2N 1N4 Canada; 2 Department of Biological Sciences, University of Alberta, Edmonton, AB T6G 2E9 Canada

**Keywords:** Physiology, Metabolism

## Abstract

Glucocorticoids (GCs) are rapidly released in response to stress and play an important role in the physiological adjustments to re-establish homeostasis. The mode of action of GCs for stress coping is mediated largely by the steroid binding to the glucocorticoid receptor (GR), a ligand-bound transcription factor, and modulating the expression of target genes. However, GCs also exert rapid actions that are independent of transcriptional regulation by modulating second messenger signaling. However, a membrane-specific protein that transduces rapid GCs signal is yet to be characterized. Here, using freshly isolated hepatocytes from rainbow trout (*Oncorhynchus mykiss*) and fura2 fluorescence microscopy, we report that stressed levels of cortisol rapidly stimulate the rise in cytosolic free calcium ([Ca^2+^]i). Pharmacological manipulations using specific extra- and intra-cellular calcium chelators, plasma membrane and endoplasmic reticulum channel blockers and receptors, indicated extracellular Ca^2+^ entry is required for the cortisol-mediated rise in ([Ca^2+^]i). Particularly, the calcium release-activated calcium (CRAC) channel gating appears to be a key target for the rapid action of cortisol in the ([Ca^2+^]i) rise in trout hepatocytes. To test this further, we carried out in silico molecular docking studies using the *Drosophila* CRAC channel modulator 1 (ORAI1) protein, the pore forming subunit of CRAC channel that is highly conserved. The result predicts a putative binding site on CRAC for cortisol to modulate channel gating, suggesting a direct, as well as an indirect regulation (by other membrane receptors) of CRAC channel gating by cortisol. Altogether, CRAC channel may be a novel cortisol-gated Ca^2+^ channel transducing rapid nongenomic signalling in hepatocytes during acute stress.

## Introduction

Glucocorticoids (GCs) are acutely released in response to stressor-mediated activation of the hypothalamus–pituitary–adrenal (HPA) axis in vertebrates^1^. GCs promote energy substrate redistribution and is essential for the metabolic adjustments to regain homeostasis in the face of stressor exposure^1^*.* This steroid action is mediated by binding to an intracellular receptor, the glucocorticoid receptor (GR), which acts as a transcription factor upon ligand-binding to regulate GC-responsive transcriptional machinery^1^. Consequently, this stress-mediated genomic effect of GCs takes longer to transpire compared to the sympathetic activation of the fight or flight response, which is rapid^2,3^. However, it is now increasingly clear that apart from the genomic responses, GCs also exert rapid effects, which are non-genomic and important in the acute stress adaptation^4–7^. For instance, studies have shown that GCs stimulate second messenger signalling, including rapid changes to intracellular cAMP and cytosolic free calcium ([Ca^2+^]i) levels, leading to downstream phosphorylation of protein kinases and the associated cellular responses^4–6^. Although, putative corticosteroid binding sites on plasma membrane were identified in vertebrates^8–10^, including teleosts^11^, as possible mediators of the rapid signaling, specific plasma membrane protein(s) transducing GC signals have yet to be discovered^4–6^.

A key mode of action for rapid physiological effects of GCs involve changes in ([Ca^2+^]i)^12,13^, and studies have shown that this steroid either stimulate or inhibit basal Ca^2+^ levels in a tissue-specific manner^6,14^. Ca^2+^ is a ubiquitous second messenger and an important regulator of the physiological actions of cells, including rapid hormonal regulation of glucose output during acute stress^15^. Rapid modulation of ([Ca^2+^]i) may be due to alterations in either the intracellular Ca^2+^ release from endoplasmic reticulum (ER) and/or extracellular Ca^2+^ entry across the plasma membrane through specific channels, including the voltage-gated calcium channels (VGCC) and/or calcium release-activated calcium (CRAC) channels^16–18^. GCs have been shown to stimulate CRAC channel gating leading to increased Ca^2+^ concentration in rat L6 muscle cells in culture^19^. Also, we recently showed that cortisol rapidly increased ([Ca^2+^]i) in the developing trunk muscle of zebrafish (*Danio rerio*), suggesting a possible role for CRAC channel activation^20^.

Liver is a key target for the metabolic actions of GCs to cope with stress, but most studies focussed on the genomic actions with little mechanistic insights into the nongenomic mode of action for this steroid^4–6,21^. In hepatocytes, a non-excitable cell model, CRAC channels play an important role in modulating cellular Ca^2+^ dynamics^17,18^. A major stimulus for CRAC channel regulation is the depletion of Ca^2+^ in the ER, which causes the sensor protein, the stromal interaction molecule (STIM), to interact with the CRAC channel modulator 1 (ORAI1) protein, the pore forming subunit of CRAC channel, and regulate channel gating^18,22,23^. Studies have shown that GCs modulation of ([Ca^2+^]i) may involve CRAC channel gating^4,6,19,20^, but this has not been studied from the standpoint of liver stress response. While cortisol rapidly increased ([Ca^2+^]i) in trout hepatocytes^4^, the mechanism leading to this rapid surge in Ca^2+^ was unknown. Also, cortisol rapidly increased the phosphorylation of protein kinase A (PKA), Akt /protein kinase B (PKB) and protein kinase C (PKC) in rainbow trout (*Oncorhynchus mykiss*) hepatocytes^24^, suggesting possible activation of second messenger signaling via putative membrane receptor(s)^4,24,25^. Given the important role of Ca^2+^ in the liver stress response^3,15^, we hypothesized that a membrane receptor transducing rapid GC signal may involve rapid modulation of ([Ca^2+^]i) in hepatocytes. Here, using freshly isolated hepatocytes from rainbow trout, we investigated whether cortisol, the principal GC in humans^1^ and teleosts^2^, directly activate plasma membrane Ca^2+^ channels to modulate ([Ca^2+^]i). GCs have been shown to either increase or decrease basal ([Ca^2+^]i) in a tissue-specific manner^6,14^, but the effect of this steroid in modulating Ca^2+^ channels activity in hepatocytes is less clear. Using targeted pharmacological approaches, including Ca^2+^ channel blockers, we investigated the mechanism by which cortisol modulates ([Ca^2+^]i) in trout hepatocytes. Our results indicate that cortisol rapidly increases ([Ca^2+^]i) in hepatocytes, and, for the first time, suggests a possible direct role for this steroid in CRAC channel gating.

## Materials and methods

### Reagents

Bovine serum albumin (BSA), Leibovitz L-15 medium were obtained from Invitrogen life technology. Collagenase (Type IV), cortisol (hydrocortisone), testosterone (4-androsten-17β**-**ol-3-one), corticosterone and β-estradiol (1,3,5[10]-estradiol-3,17 β-dio), dexamethasone, EGTA (Ethylene glycol bis (β-amino ethyl ether)-*N*,*N*,*N′*,*N′*-tetraacetic acid), nifedipine, ryanodine, trypan blue and cadmium chloride were purchased from Sigma-Aldrich (Oakville, ON, Canada). Pluoronic-F 127 (20% solution in DMSO, Molecular probes, USA), BAPTA-AM (1,2-bis o aminophenoxy) ethane-*N*,*N*,*N′*,*N′*-tetraacetic acid), Xestospongin C, H-89, U73122 and Fura 2-AM were purchased from Life Technologies, USA. Cortisol conjugated BSA was purchased from EastCoast Bio, USA. All hormones utilized during the experiment were dissolved in ethanol, the final concentration of which never exceeded 0.01%, and the control had the same amount of ethanol. All other chemicals were of analytical grade and were purchased from local suppliers.

### Animals

Juvenile rainbow trout (~ 100 to 200 g body mass) were obtained from the Allison Creek Brood Trout Hatchery Station (Crowsnest Pass, Canada) and maintained at the Animal Facility, Department of Biological Sciences, University of Calgary. The fish were maintained in a 500 L tank supplied with a constant flow of aerated de-chlorinated water with temperature maintained at 12 ± 1 °C and photoperiod set at 12 h light:12 h dark. Trout were fed commercial trout feed (Martin Mills, Elmira, ON) to satiety once daily. The fish were acclimated to this condition for at least 2 weeks, and were food deprived for 24 h prior to their use for hepatocyte isolation. All experimental protocols were approved by the Animal Care Committee at the University of Calgary and were in accordance with the Canadian Council on Animal Care guidelines, and the ARRIVE guidelines.

### Hepatocyte isolation and cell suspension

Hepatocytes were isolated following collagenase digestion as described previously^26^. In brief, fish were always sampled in the morning (0900-1000) euthanized with an overdose of 2-phenoxyethanol (600 µL/L; Fluka Analytica, USA), followed by bleeding the fish to maximize blood drain and minimize red blood cell contamination during hepatocyte isolation. The fish was dissected, and the portal vein cannulated to perfuse the liver with Medium A [(in mM), NaCl 136.9, KCL 5.4, MgSO_4_·7H_2_O 0.8, Na_2_HPO_4_·12H_2_O 0.33, KH_2_PO_4_ 0.44, HEPES 5.0, HEPES Na 5.0] for 15–20 min to remove blood, followed by Medium B (Medium A with 50 mg/100 mL collagenase; Life technology) digestion for 20–30 min. Buffers used for cannulation and enzymatic digestion were kept on ice throughout the process. After the perfusion, liver was transferred to a petri dish with Medium A, finely minced, and the suspension was filtered through two nylon filters (250 and 75 µm). The cell suspension was centrifuged 3 times (MultifugeX3R centrifuge, Thermo Scientific) at 200×*g* for 5 min at 11 °C with Medium A, after which the pellet was re-suspended in Medium C (Medium A with 1.5 mM CaCl_2_ and 1% BSA) and centrifuged as mentioned above. The pellet was re-suspended in 25 ml of L-15 medium (Leibovitz’s medium; Gibco, Thermo Scientific, USA with 5 mM NaHCO_3_ and antibiotic/antimycotic solution) and allowed to settle down on ice for 30 min. The medium was aspirated, and the settled cells were re-suspended in 10 mL of L-15 medium followed by cell counting using a haemocytometer. Cell viability was checked using trypan blue (Sigma) dye exclusion test^27^ and the viability was > 95%. The cell suspension at a concentration of 0.75 × 10^5^ million cells per ml L-15 medium were incubated in 15 mL falcon tube at 11 °C on a slow tube rotator for 24 h prior to treatments and calcium imaging.

### Cytosolic Ca^2+^ measurements

After overnight incubation cells were subjected to washing and centrifugation at 200×*g* for 5 min followed by trypan blue test. The cells were washed with Medium C and incubated with fura2-AM^28^ (10 µM in Medium C; Life technology) and 5 µL of 20% pluronic-F127 (Thermo Scientific)^29^ followed by washing and centrifugation at 200×*g* for 5 min. Then, the cell pellet was re-suspended in medium C for 30 min for the cell esterases to carry out de-esterification of fura2-AM to allow binding of fura2 to calcium, followed by imaging. Briefly, cell suspension (50 µL) was mounted on a coverslip (of approx. 18 mm) attached to a petri dish of 28 mm diameter^28^. This whole set up was then mounted on an inverted microscope (Qimaging RETIGA EXi FAST 1394) for imaging. All experiments were conducted after 30 min post-fura2-AM incubation to ensure deesterification. The hormones (or hormone agonist) added were cortisol (hydrocortisone), testosterone (4-androsten-17β**-**ol-3-one), corticosterone, 17β-estradiol and dexamethasone dissolved in 100% ethanol, with the final concentration of ethanol in all treatment groups being < 0.01%. The Ca^2+^ chelators (EGTA (2 mM)^30^, BAPTA (10 µM)^31^), plasma membrane Ca^2+^ channel inhibitors [cadmium (10 µM)^32^, Cpd5j-4 (10 µM)^33^, nifedipine (10 µM)^30^] and drugs to block intracellular pathways, including IP3 receptor [Xestospongin C (10 µM)^34^], ryanodine receptor (ryanodine(20 µM)^35^], ER Ca^2+^ ATPase [thapsigargin (10 µM)^36^), PKA (H-89 (10 µM)^37^] and phospholipase C [U73122 (10 µM)^38,39^] were added to the medium 30 min prior to cortisol addition as described previously^20^. The cortisol concentration used for the experiments were either unstressed (5 and 10 ng/mL) or physiologically stressed levels of cortisol (100 ng/mL) reported in trout. Other steroid concentrations used were all kept at the cortisol concentration (100 ng/mL) to avoid any membrane biophysical changes due to varying steroid concentrations as a factor for the observed response. ORAI-1 rabbit polyclonal antibody (1:250; Cat # 13130-1-AP, Proteintech)^40–42^ was used to block ORAI1 protein on the hepatocytes, while protein A purified mouse monoclonal cortisol antibody (1:100)^43^ was used to sequester cortisol, and anti-plant alpha tubulin antibody (1:200)^44^ was used as a control for these studies. The antibody dilutions for cortisol (1:100) and ORAI1 (1:250) were based on previous studies^43,45^. Cells were imaged immediately after cortisol and other steroid additions for 10 min, with the images captured at 10 s intervals.

Cytosolic free calcium ([Ca^2+^]i) was expressed as a ratio of fluorescence intensities at 380 nm (free of calcium) and 340 (bound to calcium). Hence, settings were made on lambda DG 4 for filters switching from 340 to 380 nm wavelengths, respectively, followed by exposure and gain settings. Light emitted from a 75-W xenon arc lamp (AH2-RX, Zeiss) passed through an excitation filter set (Chroma) to generate ultraviolet monochromatic wavelengths of 340 and 380 nm. With the aid of a computerized filter wheel (Lambda 10-2, Sutter Instruments), the cells in the chamber were alternately exposed to the two wavelengths through an objective (40×/340/0.90 N.A.). All image acquisition was computer-controlled by Northern Eclipse (EMPIX, Imaging). Images acquired were corrected for background fluorescence and shading across the field of view before calculating the ratio of the fluorescent emission intensities at each excitation wavelength (340/380 nm). Images were acquired at 10 s intervals to reduce photo bleaching. The exposure parameters for 340 and 380 nm were kept unchanged throughout the experiment. All measurements were made at room temperature (~ 20 °C).

### Validation of dye compartmentalization

To examine whether there are any calcium pockets at the intracellular level, such as in mitochondria and nucleus, which might allow an intracellular calcium release into the cytosol, Triton X-100 was used to determine any possible compartmentalization of fura2^46^. Triton X-100 permeabilized hepatocyte cell membrane and organelles that releases trapped Ca^2+^ inside the organelles after fura2 incubation. Dye compartmentalization was a confirmatory step to determine that no residual fura2 were trapped in the organelles apart from the cytosol (Fig. S3). Our ratiometric recordings of Ca^2+^ bound and Ca^2+^ free form showed no significant rise in calcium after triton treatment indicating a lack of dye compartmentalization within the cell.

### Immunofluorescent labelling

Isolated hepatocytes were cultured on 22 mm coverslips in 6 well plates (Sarstedt, Inc. USA) for 24 h prior to treatment exposure. After exposure to cortisol (100 ng/mL) for 5 min, the cells were fixed using ice cold methanol and stored at 4 °C. The coverslips with the fixed cells were tapped on paper towel to remove excess methanol followed by air-drying. Hydrophobic pen was used to draw a barrier around the coverslip boundary to secure the central area for antibody application. Coverslips with cells attached were then incubated with a permeabilization buffer (0.1%TritonX-100 in Medium A) for 10 min followed by washing with Medium A (with 0.1%Tween-20) 3 times at 5 min interval. The permeabilized cells were incubated with a blocking solution (Medium A with 2% BSA) for 3 h at 37 °C followed by overnight incubation with anti-ORAI antibody at 4 °C. The next day cells were then probed with the secondary antibody, which consisted of goat anti-rabbit Alexa 488 conjugate (1:500{GREEN}; Thermo Fisher Scientific, CA). Double antibody staining was carried out by incubating the cells with caveolin-1 mouse monoclonal IgG_2B_ antibody (1:500; sc-53564: Santa Cruz Biotechnology) for overnight at 4 °C. Both the ORAI and caveolin-1 antibodies have been tested in zebrafish previously^20^. Secondary antibody donkey anti-mouse Alexa 594 conjugate (1:500{RED}; Thermo Fisher Scientific, CA) was carried out the next day after washing. DAPI (100 ng/mL) staining was done to identify nuclear localization prior to mounting. Coverslips were mounted to clean slides using DABCO (Antifade reagent, Sigma Aldrich, CAN) followed by sealing the periphery using transparent nail paint. This whole mounting step lasts for an hour to air dry. Post drying, slides were wrapped in aluminium foil and stored at 4 °C until imaging. This step is crucial in keeping the staining stable for longer period with less photo bleaching.

### Western blotting

Trout liver homogenate for western blotting was prepared as described previously^26^. Whole trout liver was dissected out from terminally anesthetised trout. The liver was homogenized in TCD buffer (300 mM sucrose, 10 mM TRIS–HCL, 1 mM DTT, 0.5 mM CaCl_2_, Roche protease (Roche Diagnostics, CAN), and the homogenized mixture was subjected to multiple centrifugation steps at 4 °C for separation of cellular fractions as described previously^47^. The membrane fractions of trout liver were prepared using ultracentrifugation at 100,000×*g* as described previously^26^. Membrane fraction enrichment and any cytosolic contamination was detected by measuring the activities of 5′AMP nucleotidase^48^ and lactate dehydrogenase (LDH)^26,47,49^.

Protein concentration was measured using bicinchoninic acid (BCA) method. Membrane fractions and liver homogenate were diluted in Laemmli’s buffer [0.06MTRIS-HCL (pH6.8), 20%(V/V) glycerol, 0.02% (w/v) SDS, 0.025%(w/v) bromophenol blue, 5% β mercaptoethanol]. A total of 40 μg protein lysate was resolved on a 10% SDS-PAGE, transferred onto a 0.45 μm nitrocellulose membrane (Biorad, CAN)^50^ as described previously^47^. At room temperature, membranes were blocked for 1 h in 5% skim milk, followed by an overnight incubation with rabbit polyclonal ORAI-1primary antibody (1:250) at 4 °C. Membrane were further washed and incubated with secondary goat anti rabbit IgG (H+L) horse radish peroxidase (HRP conjugate; BIORAD # 1706515) (1:3300 diluted in 5% skim milk) for 1 h at room temperature. Blots were washed followed by protein band detection using chemiluminiscence substrate ECLplus incubation according to manufacturer’s instructions and imaged using Syngene G-Box Imager (Syngene, USA).

### In silico structural modelling

The docking was done using AutoDock Vina v1.1 program. Input files for AutoDock Vina were prepared using AutoDock tools (The Scripps Research Institute, La Jolla, CA, USA). File preparation involved changing atom type, removal of water molecules and addition of polar hydrogen atoms. Structure files were saved in PDBQT format. CRAC channel structure (X-ray crystal structure of *Drosophila melanogaster*) was downloaded from RCSB protein data back in PDBQT format. Further, metal ions were removed from the structure to avoid insignificant binding. Polar hydrogens were added to the PDB file using AutoDockTools and converted to PDBQT file. Hydrogen addition mimics a more realistic environment for docking. To find the entire binding site on CRAC, the grid was modified to cover the entire protein (CRAC), as we are unaware of the binding sites. The ligands, including cortisol, corticosterone, estradiol, testosterone and dexamethasone were used from ZINC database^51^. The *Drosophila melanogaster* CRAC channel (4HKR.pdb) was used as a receptor. The receptor protein coordinates of CRAC channel with PDB id of 4HKR.pdb were considered to study binding sites. The structural integrity of the binding was assessed by analyzing the root mean square deviation (RMSD) between interacting molecules. RMSD values are used to validate protein–ligand binding in terms of binding energy and interaction established between protein and ligand. CRAC channel structure is a hexameric assembly of four transmembrane helices (M1–M4) and helix extension of M4 extending into the cytosol. The channel pore is made up of six M1 helices to form the inner pore. M2 and M3 together form the outer lining for M1 helices and separate them from M4 helices. M4 helices are the peripheral outer ring subunit of CRAC that interacts with STIM for channel gating. Studies confirm that STIM binding to Leu^319^ or Ile^319^ at the M4 extension A and B is critical for channel activation. The best results containing 8 coordinates of each ligand (as mentioned above) are considered for further analysis. PyMOL was used to predict the orientation of amino acids. The metal ions, known to bind the core of the receptor, were removed at the beginning of the docking process to avoid non-specific binding predictions. The exhaustiveness value was set to default (8) and a local computer (with 8 core CPUs) was used for the docking. During the docking process, the receptor was treated as a rigid molecule and the ligands were flexible in the binding site. One single best score-binding site of each ligand was considered for further analysis.

### Statistics analysis

The values are presented as the mean ± SEM. Data were analyzed using student t-test or one-way ANOVA followed by a post hoc Holm Sidak test. Equal variance was tested using levene median test and normality was tested using Shapiro wilk test. A p value < 0.05 was considered significant.

## Results and discussion

GCs are key stress hormones and are important in re-establishing homeostasis after stressor exposure^2,52^. However, most of the stress coping actions of GCs have been attributed to its binding to GR, a ligand-bound transcription factor, and regulating target genes, including encoding proteins involved in metabolism and immune function^53^. Calcium is a key second messenger that is an important mediator of the cellular stress response^54^. Hormonal effects on rapid intracellular calcium modulation during stress has been shown in several cell types, including hepatocytes^3^. Here we are showing a role for GCs in rapidly increasing ([Ca^2+^]i), and this may be a mechanism for the rapid nongenomic actions of GCs on liver function^4,5,24^. Our results for the first time suggest CRAC channels as a potential cortisol-gated calcium channel in trout hepatocytes.

### Cortisol rapidly stimulates ([Ca^2+^]i)

**Figure 1:**
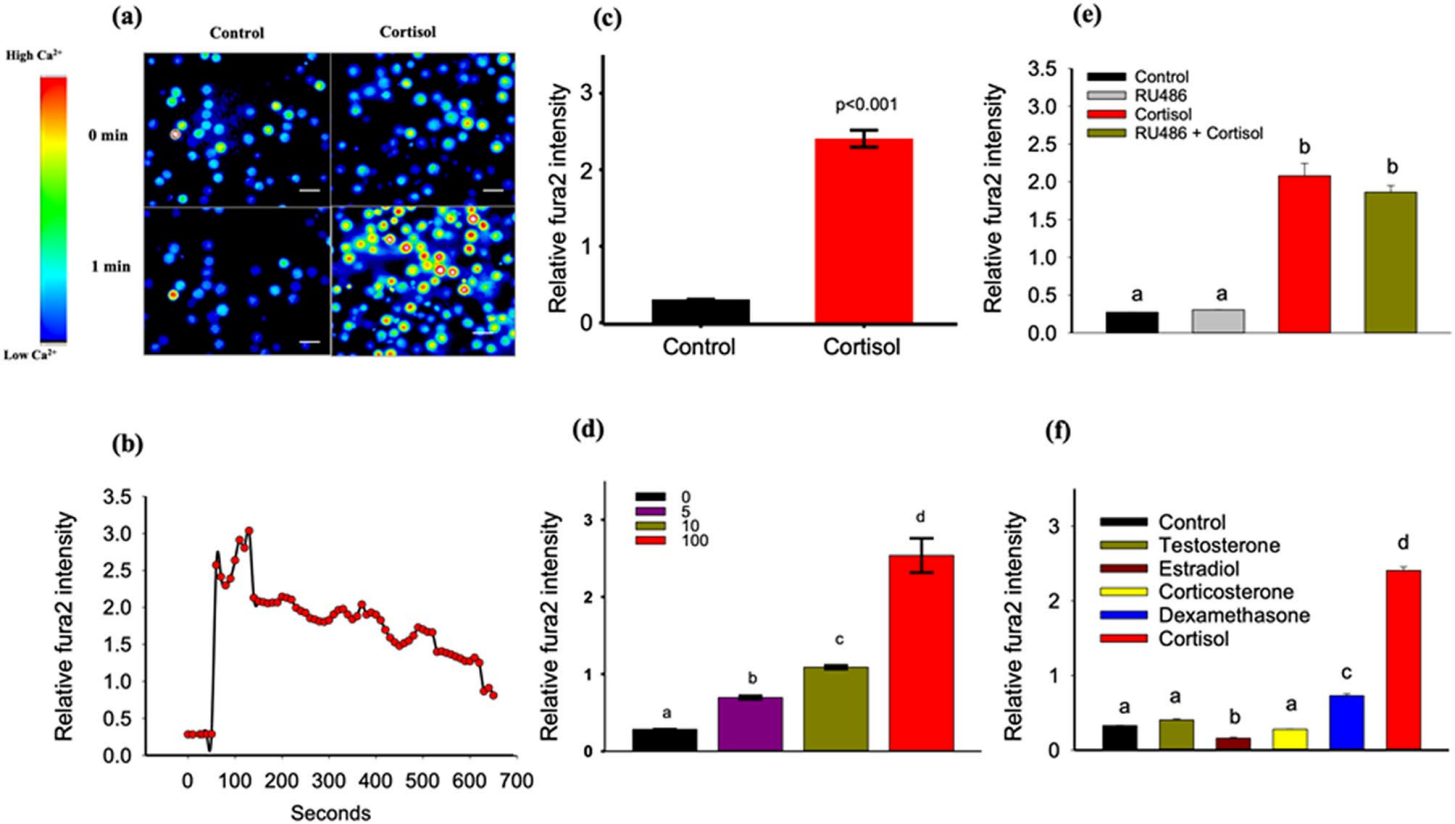
Cortisol rapidly stimulates ([Ca^2+^]i). Hepatocytes incubated with ratiometric dye fura2 and with and without cortisol for 1 min. Enhanced dye intensity with cortisol was seen in > 75% of hepatocytes; scale bar represents an approximate cell diameter of 25 μm (field of view at 40x has1392 × 1040 pixel with 6.19pixel/μm) (**a**) Representative fluorescence images taken at 0 or 1 min either with or without the addition of cortisol. (**b**) Representative line graph showing the time-dependent fura2 intensity ([Ca^2+^]i) changes in response to cortisol exposure. (**c**) Bar graph showing ([Ca^2+^]i) changes at 60 s after cortisol addition (t test; p < 0.001). (**d**) Dose-related ([Ca^2+^]i) response at 60 s to cortisol (0, 5,10 and 100 ng/ml). (**e**) Bar graph showing ([Ca^2+^]i) changes at 60 s in response to either control, RU486, cortisol or cortisol + RU486. (**f**) Bar graph showing ([Ca^2+^]i) changes at 60 s in response to either control, testosterone, estradiol, corticosterone, dexamethasone and cortisol exposure. All bars represent mean ± SEM (n = 5–6 fish; ~ 100 cells from each fish); Bars with different letters are significantly different (one-way ANOVA; p < 0.05).

Using fura2 methods we show that stressed levels of cortisol (100 ng/mL) rapidly increases ([Ca^2+^]i) in isolated hepatocytes (Fig. 1a). This Ca^2+^ wave tapers over a 10 min period (Fig. 1b), and was repeatable by cortisol stimulation after a washout (Fig. S1a). The rise in ([Ca^2+^]i) was significantly greater (p < 0.001) with stress levels (100 ng/mL) of cortisol (Fig. 1c) compared to the unstressed levels (5 and 10 ng/mL; Fig. 1d) in trout. These ([Ca^2+^]i) responses were also mimicked by the membrane impermeable cortisol-BSA (Figs. S1b,c), supporting the idea of a membrane-mediated rapid signalling. Pre-treatment with the GR-inhibitor RU486 failed to block the cortisol-induced ([Ca^2+^]i) wave (Fig. 1e), suggesting little involvement of the classical intracellular GR. While steroids, including testosterone, estradiol and aldosterone modulate ([Ca^2+^]i) in different cell types^6,21^ our results indicate the greatest ([Ca^2+^]i) increase with cortisol (> 60%) in hepatocytes compared to the other steroids tested (Fig. 1f).

### Pharmacological characterization of the source of calcium

**Figure 2:**
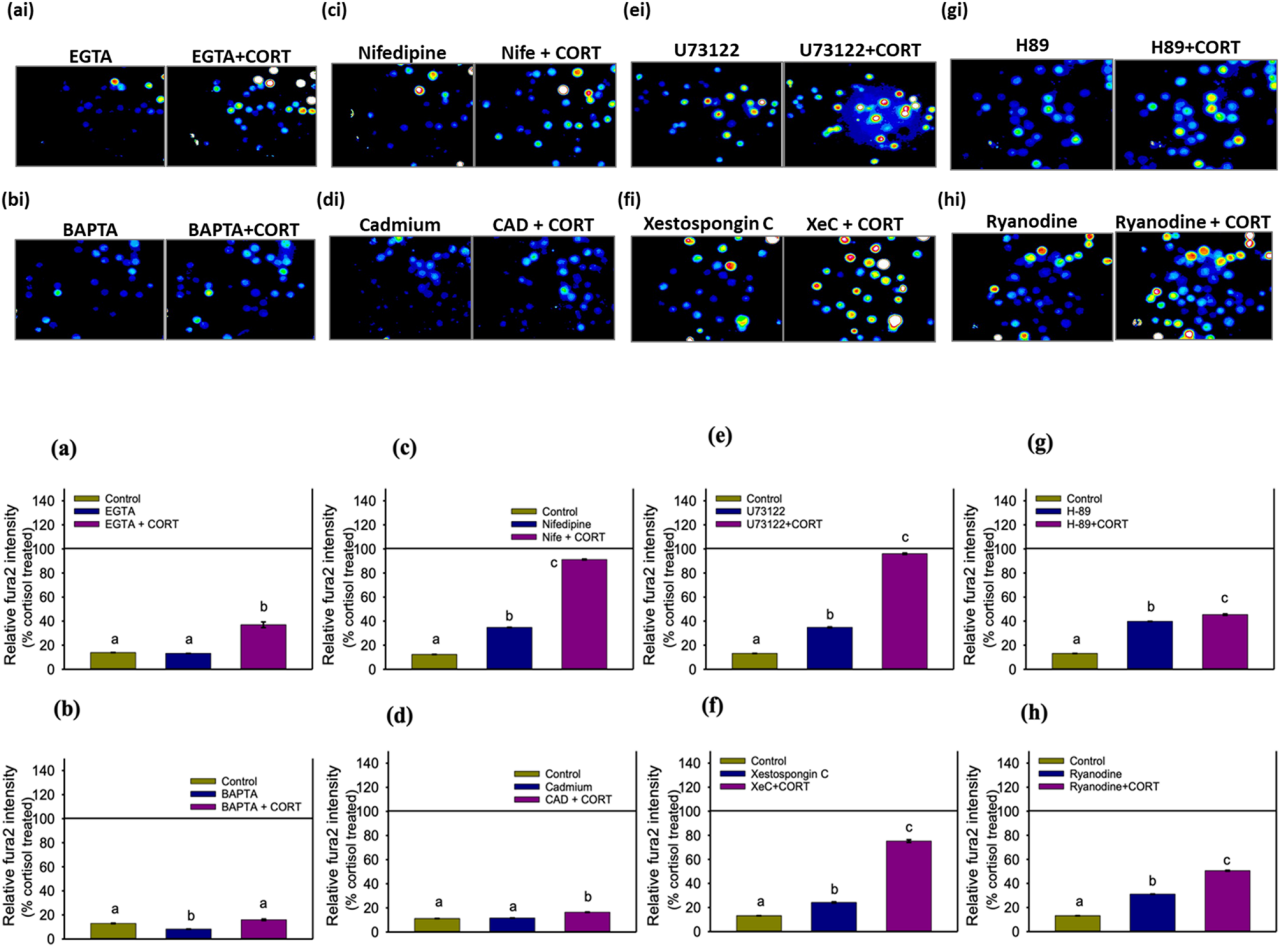
Pharmacological characterization of the source of calcium. Hepatocytes were treated with different blocking agents and chelators 30 min prior to cortisol addition. All results are shown as % of cortisol (100 ng/ml)-treated fura2 intensity, which is represented by the horizontal line at 100%. Representative fluorescence images taken at 1 min either with or without the addition of cortisol are shown above (ai–hi), while the corresponding quantification is shown below as a bar graph (**a**–**h**). Bar graph showing fura2 intensity ([Ca^2+^]i) changes at 60 s relative to cortisol treatment with (**a**) either control, EGTA or EGTA + Cortisol, (**b**) either control, BAPTA or BAPTA + Cortisol, (**c**) either control, Nifedipine (Nife) or Nife + Cortisol, (**d**) either control, cadmium (CAD) or CAD + Cortisol, (**e**) either control, U73122 or U73122 + Cortisol, (**f**) either control, xestospongin C (XeC) or XeC + Cortisol, (**g**) either control, H-89 or H-89 + Cortisol, (**h**) either control, ryanodine or ryanodine + Cortisol. All bars represent mean ± SEM (n = 5–6 independent fish; ~ 100 cells from each fish); bars with different letters are significantly different (one-way ANOVA; p < 0.05).

While several studies have showed GCs to modulate intracellular calcium levels^6^, no study has actually investigated in-depth whether the origin of the calcium is from extracellular and/or intracellular stores in hepatocytes. In teleosts, one other study suggested VGCC as a possible target for cortisol-mediated rapid inhibitory effect on prolactin secretion in dispersed pituitary cells of the tilapia, *Oreochromis mossambicus*^55^. Here, we asked whether extracellular [Ca^2+^] contributed to the ([Ca^2+^]i) rise by manipulating the extracellular Ca^2+^ availability. In the absence of extracellular Ca^2+^ cortisol did not elicit a ([Ca^2+^]i) rise in trout hepatocytes (Fig. S1d). While EGTA significantly reduced (> 60%) cortisol-induced ([Ca^2+^]i) rise (Fig. 2a), a modest (~ 20%) increase in ([Ca^2+^]i) compared to the control suggests that cortisol may stimulate Ca^2+^ release also from ER (Fig. 2a). This was further confirmed by complete abolishment of cortisol-stimulated ([Ca^2+^]i) increase in cells pre-treated with the membrane permeant Ca^2+^ chelator BAPTA-AM (Fig. 2b). Hepatocytes are non-excitable cells and rely less on voltage-gated Ca^2+^ channels (VGCC) and more on CRAC channel for hormone-mediated ([Ca^2+^]i) modulation^17,18^. To test this, we treated hepatocytes with the L-type VGCC inhibitor nifedipine, which blocked only 10% of the cortisol-induced ([Ca^2+^]i) response (Fig. 2c), suggesting very little involvement of VGCC in cortisol-mediated ([Ca^2+^]i) rise. However, cadmium, a non-specific blocker of both VGCC and CRACC^56^, completely abolished cortisol-induced ([Ca^2+^]i) rise (Fig. 2d) suggesting a role of CRAC channel in the observed cortisol response. CRAC channel gating occurs in response to calcium depletion in the ER, which causes the STIM to interact with the ORAI1 protein leading to CRAC channel gating^18^. Therefore, we tested the involvement of cortisol in the depletion of ER Ca^2+^ stores using a combination of PLC (U73122), PKA (H-89), inositol 1,4,5, triphosphate receptor (IP3R) (Xestospongin C) and RYR (ryanodine) inhibitors. While the inhibition of the PLC-IP3R and PKA-RYR pathways blocked cortisol-induced biphasic ([Ca^2+^]i) rise by only 5–20% (Fig. 2e,f) and ~ 50% (Fig. 2g,h), respectively, none of these completely abolished the cortisol-induced ([Ca^2+^]i) rise. These results led us to propose that activation of PKA-RYR pathway may be one possible mechanism by which cortisol rapidly stimulates ([Ca^2+^]i) rise in trout hepatocytes. However, the plasma membrane receptor involved in this activation by cortisol remains elusive. Also, whether activation of the PKA-RYR pathway partly accounts for the CRAC gating by cortisol remains to be determined.

### Cortisol stimulates Ca^2+^ entry via CRAC channel

**Figure 3:**
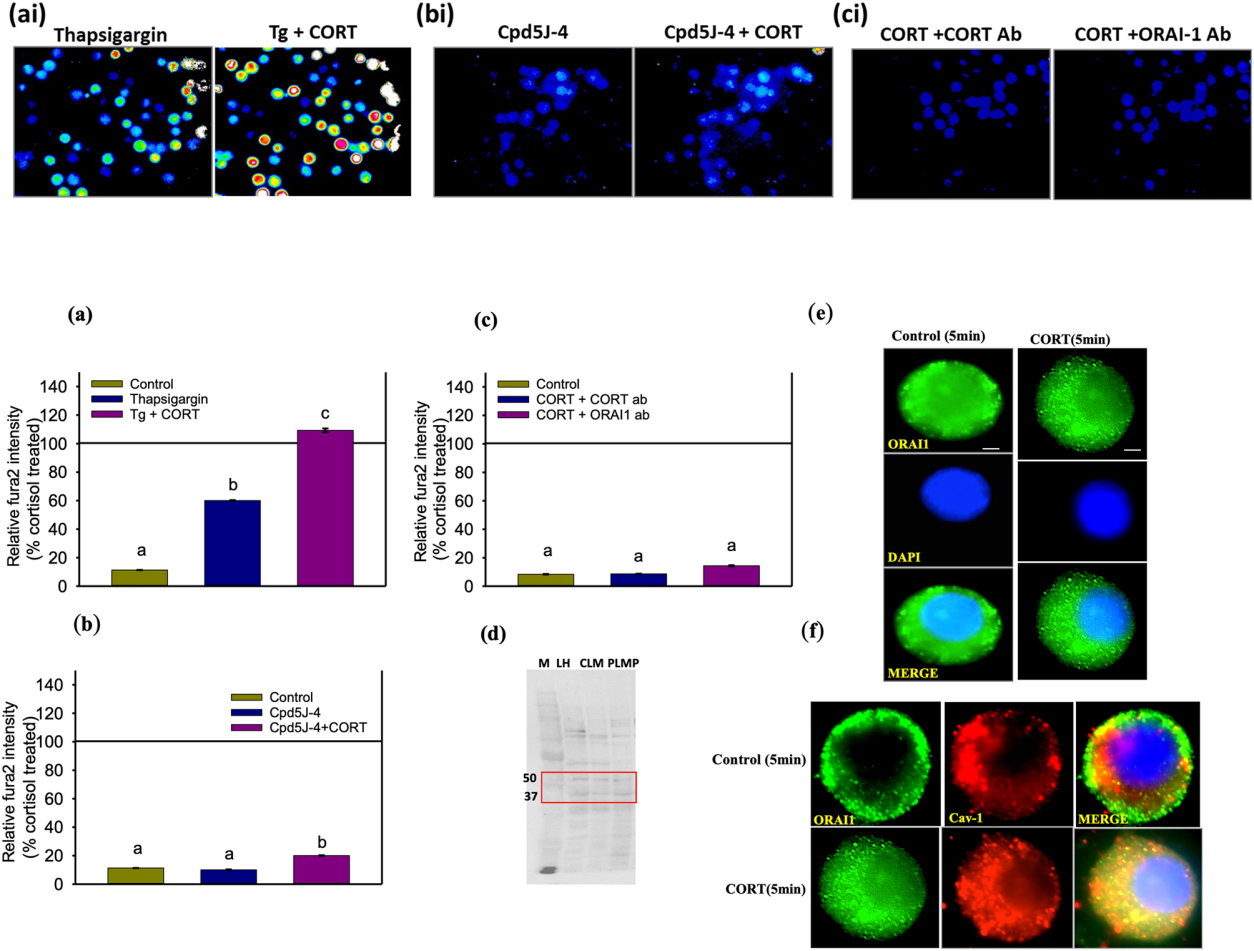
Cortisol stimulates Ca^2+^ entry via CRAC channel. (**a**) Results are shown as % of cortisol-treated fura2 intensity, which is represented by the horizontal line at 100%. Representative fluorescence images taken at 1 min either with or without the addition of cortisol are shown above (ai–ci), while the corresponding quantification is shown below as a bar graph (**a**–**c**). Bar graph showing fura2 intensity ([Ca^2+^]i) changes in hepatocytes at 60 s relative to cortisol treatment with either control, thapsigargin or Tg + Cortisol. (**b**) Bar graph showing fura2 intensity ([Ca^2+^]i) changes in hepatocytes at 60 s relative to cortisol treatment with either control, Cpd5J-4 or Cpd5j-4 + Cortisol. (**c**) Bar graph showing ([Ca^2+^]i) changes in hepatocytes at 60 s relative to cortisol treatment with either control, Cortisol exposure to cells treated with cortisol antibody (1:100) or Cortisol exposure to cells treated with ORAI1 antibody (1:250) (**d**) ORAI-1expression (~ 51 Kda) was detected in the crude liver membrane (CLM), plasma membrane purified (PLMP) and liver homogenate (LH) of trout using western blotting. See Figure S4 for the image of the blot with the molecular marker. (**e**) Representative immunofluorescence localization of ORAI1 in control and cortisol-treated (5 min) hepatocytes. The top panel shows increase ORAI1 expression on the surface of cortisol treated cell relative to the control (diffused distribution). The middle panel shows DAPI staining (nucleus) and the lower panel shows a merged image. (**f**) Colocalization of ORAI-1 with Caveolin-1 in control cells and cells treated with cortisol for 5 min. Colocalization was confirmed by IMAGE J- colocalization analyzer tool. Green indicates ORAI1 expression, while the red shows Caveolin-1 expression. The merged image showing yellow colour represents colocalization, and it is higher in the cortisol hepatocytes compared to the control. All bars represent mean ± SEM (n = 5–6 independent fish); Bars with different letters are significantly different (one-way ANOVA; p < 0.05). Image scale bar represents an approximate cell diameter of 25 μm (field of view at 100x has 650 × 520 pixel with 7.7 pixel/μm).

To test whether cortisol might stimulate CRAC channel gating, we first depleted the internal Ca^2+^ stores with thapsigargin (Tg), a sarco/endoplasmic reticulum calcium ATPase (SERCA) inhibitor, that facilitates STIM-mediated CRAC channel gating^17^. As expected, Tg treatment induced an increase in ([Ca^2+^]i) (Fig. 3a), supporting CRAC channel gating in trout hepatocytes^17^. However, this response was significantly greater in the presence of cortisol (Fig. 3a) suggesting direct modulation of CRAC channel by this steroid. To test this, we treated hepatocytes with the ORAI1/CRACC blocker Cpd5j-4 and then subjected the cells to an acute cortisol stimulation. Cpd5j-4 completely abolished the cortisol-induced rapid ([Ca^2+^]i) rise in hepatocytes (Fig. 3b), and this response was similar to that seen with cadmium (Fig. 2d), reinforcing the idea that cortisol may stimulate CRAC channel gating. To further confirm this, hepatocytes were pre-exposed to either cortisol or ORAI1 function-blocking antibodies before cortisol addition. Immunoneutralization completely abolished the cortisol-induced ([Ca^2+^]i) rise (Fig. 3c), underpinning a key mechanistic role for ORAI1 in regulating Ca^2+^ entry by cortisol. We confirmed ORAI1 (~ 51 kDa) expression in trout liver (Fig. 3d**)**, and cortisol stimulation rapidly recruited ORAI1 to the membrane (Fig. 3e). The greater colocalization of ORAI1 with cav-1^57^ supports a rapid cortisol-mediated recruitment to the plasma membrane (Fig. 3f), and this may play a role in the cortisol-mediated maintenance of ([Ca^2+^]i) wave in hepatocytes (Fig. 1b).

### Predicted cortisol-binding site on CRAC channel

**Figure 4:**
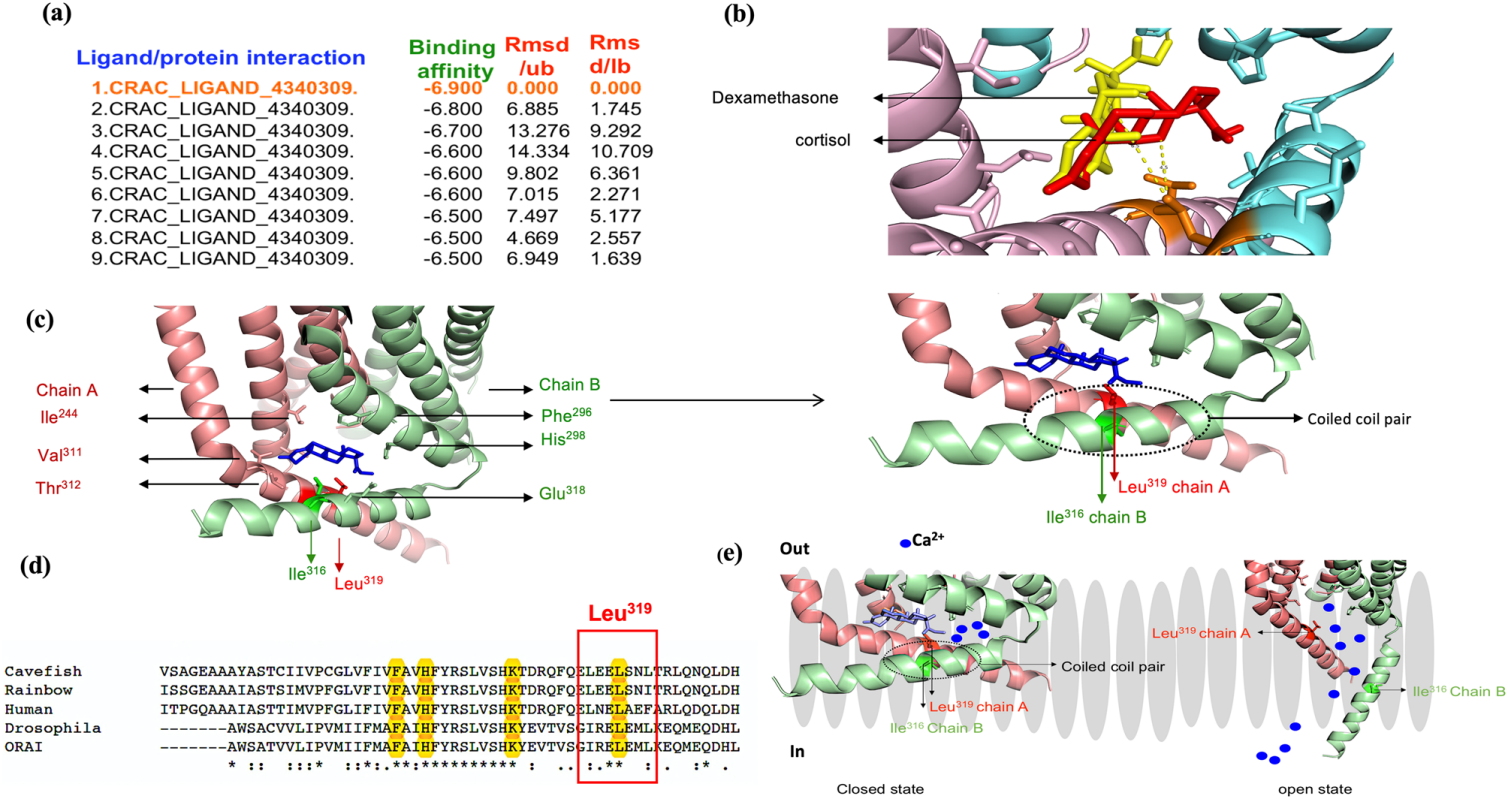
Predicted cortisol-binding site on CRAC channel. Predicted interaction sites of cortisol with CRAC channel from molecular docking. (**a**) Binding energies and the root mean square deviation (RMSD) scores of the best possible clusters from the docking results of cortisol interaction with CRAC channel. The most favourable binding (in orange) is considered for understanding the gating mechanism based on the docking score. (**b**) The interaction site of cortisol (red) (distance ~ 4.6 Å) and dexamethasone (yellow) (distance ~ 6.5 Å) at TM4 from the active site of chain B (cyan). The smaller distance of cortisol from the interaction site indicates its larger influence on active site. (**c**) (Left) Cortisol interacting residues on CRAC chain A (light red) and B (light green) within proximity (distance ~ 4.5 Å) are shown in sticks. Cortisol is shown in blue. (Right) A closed view of the coiled-coil pair formation in closed state of the CRAC channel due to the interaction of Leu^319^ and Ile^316^ of both the chains and their presence within proximity of cortisol. (**d**) Sequence alignment showing the location of some of the conserved residues including active site Leu^319^ at TM3 and TM4 helices. (**e**) Schematic representation of cortisol involvement in CRAC gating. The membrane is shown in gray. The presence of coiled-coil pair in closed state (left) and opening of the channel in its absence (right). Cortisol binding to the coiled residues Leu^319^ or Ileu^316^ may potentially unlatch the coiled structure similar to STIM and allow Ca^2+^ gating by decreasing the energy barrier and increasing Ca^2+^selectivity of CRAC. *iCa*^*2*+^ intracellular calcium, *CRAC* calcium release activated channel, Transmembrane helix 4(TM4).

**Figure 5:**
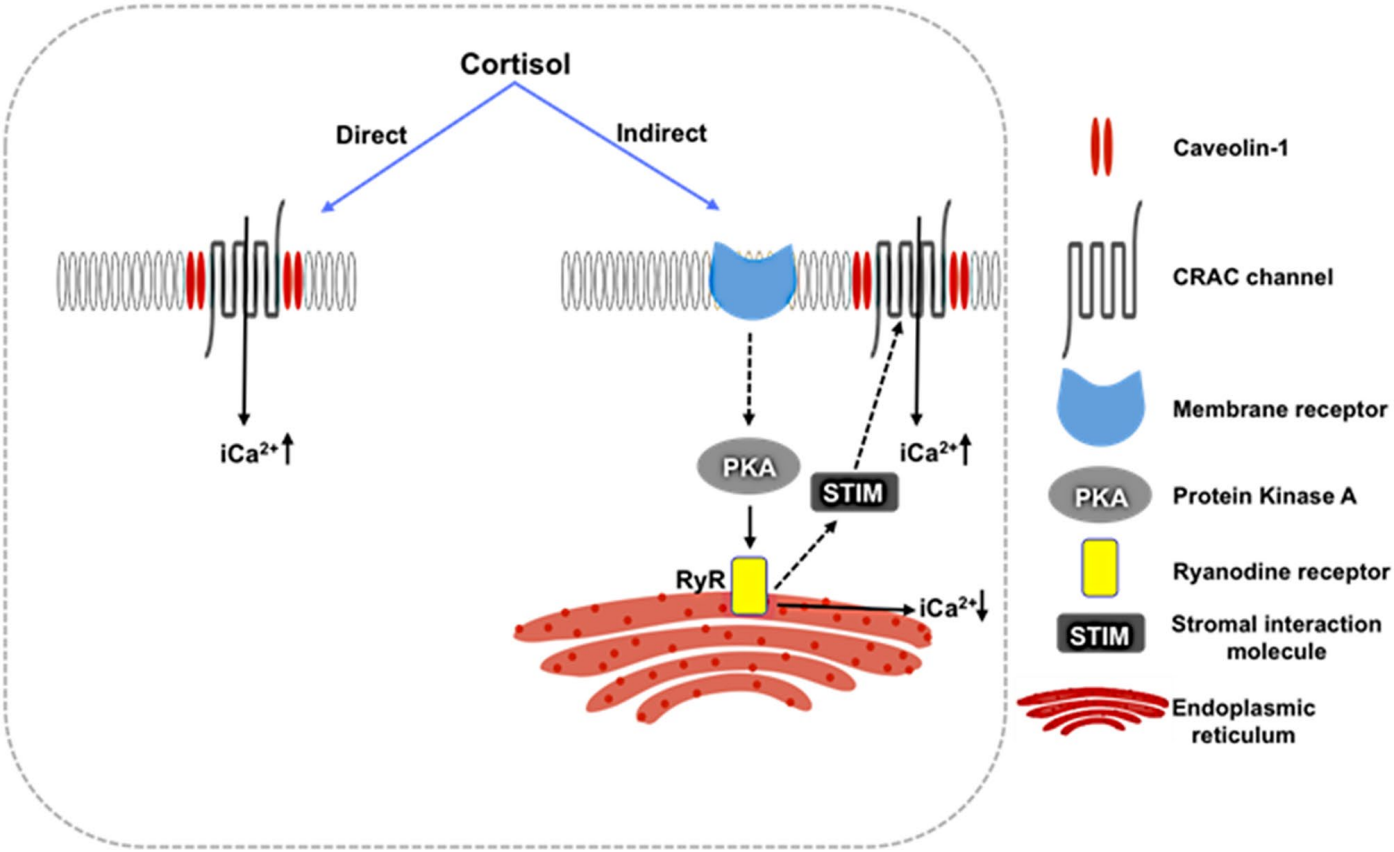
A hypothetical model for rapid cortisol stimulation of ([Ca^2+^]i) in hepatocytes. We propose two modes of action: (1) an indirect effect of cortisol by activating yet unknown membrane receptors to deplete endoplasmic reticulum stores, which then results in the interaction of STIM with ORAI1 to open the CRAC channels causing a biphasic increase in ([Ca^2+^]i). Our results suggest that PKA-RYR pathway may be more important than the PLC-IP3R pathway in contributing to this indirect cortisol-mediated CRAC channel gating in hepatocytes. (2) A direct effect of cortisol by binding to the ORAI1 and opening the pore to allow extracellular calcium. Based on our molecular simulation, and pharmacological approach, we propose that CRAC channels may act as cortisol-gated ion channels in hepatocytes.

To explore the possibility that cortisol binding to CRAC channel as a possible mechanism for rapid ([Ca^2+^]i) rise, we carried out in silico molecular docking studies using the *Drosophila* ORAI (4HKR.pdb)^58^. While modulation of ion channel activity by steroids have been studied^60–61^, no study has looked at the CRAC channel activation. Here, our modelling predicts a putative cortisol-binding site in the ORAI gating domain (Fig. 4a, Fig. S2a,b). Although best-docking coordinates predicts cortisol and dexamethasone to be most favourable to bind ORAI1 (Fig. 4b), the larger distance of dexamethasone (6.5 Å) relative to cortisol (4.5 Å) from the cavity residues may limit its ability to facilitate channel gating (Fig. 4b). Indeed, dexamethasone treatment produced < 60% of the cortisol-induced ([Ca^2+^]i) rise in hepatocytes (Fig. 2f). Cortisol showed interaction with Ile^316^ and Leu^319^ residues of chain A and B of M4 helix^62,63^ (Fig. 4c), which in the closed conformation interact with one another to form a hydrophobic patch, leading to an antiparallel coiled-coil structure (Fig. 4c). Our docking simulation predicted that cortisol binding to the Leu^319^ may disrupt the Ile^316^ and Leu^319^ link and open the channel, similar to the conformational change associated with STIM binding^62,63^. Sequence alignment (Clustal Omega, Uniprot) showed that the interacting amino-acid residues of ORAI1 are conserved across species (Fig. 4d), and the strong cortisol binding prediction to the STIM binding site, suggests this as a possible mechanism for cortisol-induced CRAC channel gating (Figs. S2c). Docking of cortisol was also performed with the recent model of both closed and open CRAC–ORAI structure^64^, and cortisol was able to bind to the STIM binding site only when the channel was in a closed state, while the steroid loses the binding pocket when the channel is in an open confirmation (Fig. 4e**)**. These results underscore a potential direct modulation of ORAI1 by cortisol, and complements the evidence provided by the pharmacological manipulation (Figs. 1, 2, 3), directing to a cortisol-stimulated CRAC channel gating. It is important to note that direct CRAC channel activation may be one possible mechanism by which cortisol stimulates channel gating to initiate the rapid nongenomic signalling. However, we cannot rule out other possible mechanisms, including other membrane receptor(s)^5,8,10,11^ that may activate channel opening indirectly by depleting ER calcium stores (Fig. 5). The lack of STIM antibody for our cell model prevented us from testing this further.

## Conclusions and outlook

Our results point to a rapid effect of cortisol on CRAC channel gating as a mechanism for the nongenomic cortisol action on hepatocyte stress response^11,24^. We propose two modes of action of cortisol in regulating CRAC channel gating (Fig. 5). (1) An indirect effect of cortisol by activating a yet unknown membrane receptor to deplete ER stores, resulting in the interaction of STIM with ORAI1 to open CRAC channels and causing an increases in ([Ca^2+^]i). While studies have shown that the PLC-IP3R pathway is a major player in increasing ER store depletion and CRAC channel gating^17^, our results suggest that PKA-RYR pathway may be more important in modulating CRAC channel opening in trout hepatocytes under short-term stress. However, the membrane protein(s) involved in transducing the GC signal is currently unknown. (2) A direct effect of cortisol by binding to the ORAI1 leading to CRAC channel gating causing the ([Ca^2+^]i) wave in trout hepatocytes. Based on our molecular simulation, and pharmacological approach, we propose that CRAC channels may function as a cortisol-gated Ca^2+^ channel during stress in hepatocytes. To confirm this, future studies should focus on assessing the binding characteristics of cortisol on ORAI1 proteins.

The CRAC channels are not just limited to hepatocytes, but also are prevalent in other non-excitable cells, including immune cells^18^. So the results may have far ranging implications, especially considering that GCs are widely used as therapeutics for inflammation and other immune-related disorders involving Ca^2+^ signalling^6,65^. Chronic usage of this steroid leads to multiple side effects because of its predominant genomic actions, including osteoporosis, fluid and mineral imbalance and Type 2 diabetes^65^. Our in silico determination of a binding site for cortisol regulating pore opening on CRAC channel has potential therapeutic implication in the development of drugs that specifically target this binding pocket, thereby offsetting any unwanted side effects associated with GR activation due to chronic corticosteroid use^6^. Also, the novel role of CRAC channel as a possible GC-gated calcium channel has relevance as a potential therapeutic target for the treatment of stress-related disorders, including ER stress, major depressive disorders and immune dysfunctions^15,66^, all of which are associated with abnormal Ca^2+^ dynamics, and warrants further investigations.

## Supplementary Information

Supplementary Information 1.

## References

[1] Nicolaides NC, Kyratzi E, Lamprokostopoulou A, Chrousos GP, Charmandari E (2015). Stress, the stress system and the role of glucocorticoids. NeuroImmunoModulation.

[2] Mommsen TP, Vijayan MM, Moon TW (1999). Cortisol in teleosts: Dynamics, mechanisms of action, and metabolic regulation. Rev. Fish Biol. Fish..

[3] Fabbri E, Moon TW (2016). Adrenergic signaling in teleost fish liver, a challenging path. Comp. Biochem. Physiol. Part B Biochem. Mol. Biol..

[4] Das C, Thraya M, Vijayan MM (2018). Nongenomic cortisol signaling in fish. Gen. Comp. Endocrinol..

[5] Johnstone WM, Honeycutt JL, Deck CA, Borski RJ (2019). Nongenomic glucocorticoid effects and their mechanisms of action in vertebrates. Int. Rev. Cell Mol. Biol..

[6] Panettieri RA, Schaafsma D, Amrani Y, Koziol-White C, Ostrom R, Tliba O (2019). Non-genomic effects of glucocorticoids: An updated view. Trends Pharmacol. Sci..

[7] Losel RM, Falkenstein E, Feuring M, Schultz A, Tillmann H-C, Rossol-Haseroth K, Wehling M (2003). Nongenomic steroid action: Controversies, questions, and answers. Physiol. Rev..

[8] Orchinik M, Murray TF, Moore FL (1991). A corticosteroid receptor in neuronal membranes. Science (80–).

[9] Orchinik M, Matthews L, Gasser PJ (2000). Distinct specificity for corticosteroid binding sites in amphibian cytosol, neuronal membranes, and plasma. Gen. Comp. Endocrinol..

[10] Breuner CW, Orchinik M (2009). Pharmacological characterization of intracellular, membrane, and plasma binding sites for corticosterone in house sparrows. Gen. Comp. Endocrinol..

[11] Johnstone WM, Mills KA, Alyea RA, Thomas P, Borski RJ (2013). Characterization of membrane receptor binding activity for cortisol in the liver and kidney of the euryhaline teleost, Mozambique tilapia (*Oreochromis mossambicus*). Gen. Comp. Endocrinol..

[12] Kim M, Lee G, Jung E, Choi K, Oh G, Jeung E (2009). Dexamethasone differentially regulates renal and duodenal calcium-processing genes in calbindin-D9k and-D28k knockout mice. Exp. Physiol..

[13] Chen S, Wang X, Zhang X, Wang W, Liu D, Long Z, Dai W, Chen Q, Xu M, Zhou J (2011). High-dose glucocorticoids induce decreases calcium in hypothalamus neurons via plasma membrane Ca^2+^ pumps. NeuroReport.

[14] Joëls M, Karst H (2012). Corticosteroid effects on calcium signaling in limbic neurons. Cell Calcium.

[15] Amaya MJ, Nathanson MH (2013). Calcium signaling in the liver. Compr. Physiol..

[16] Berridge MJ, Bootman MD, Roderick HL (2003). Calcium signalling: Dynamics, homeostasis and remodelling. Nat. Rev. Mol. Cell Biol..

[17] Parekh, A. B., & Putney, J. W. Store-operated calcium channels. *Physiol. Rev*. **85**, 757–810 (2005). http://physrev.physiology.org/content/85/2/757.abstract. 10.1152/physrev.00057.2003 15788710

[18] Prakriya M, Lewis RS (2015). Store-operated calcium channels. Physiol. Rev..

[19] Itagaki K, Menconi M, Antoniu B, Zhang Q, Gonnella P, Soybel D, Hauser C, Hasselgren P-O (2010). Dexamethasone stimulates store-operated calcium entry and protein degradation in cultured L6 myotubes through a phospholipase A2-dependent mechanism. Am. J. Physiol. Physiol..

[20] Das C, Faught E, Vijayan MM (2020). Cortisol rapidly stimulates calcium waves in the developing trunk muscle of zebrafish. Mol. Cell. Endocrinol..

[21] Lösel R, Wehling M (2003). Nongenomic actions of steroid hormones. Nat. Rev. Mol. Cell Biol..

[22] Feske S, Gwack Y, Prakriya M, Srikanth S, Puppel S-H, Tanasa B, Hogan PG, Lewis RS, Daly M, Rao A (2006). A mutation in Orai1 causes immune deficiency by abrogating CRAC channel function. Nature.

[23] Prakriya M, Feske S, Gwack Y, Srikanth S, Rao A, Hogan PG (2006). Orai1 is an essential pore subunit of the CRAC channel. Nature.

[24] Dindia L, Murray J, Faught E, Davis TL, Leonenko Z, Vijayan MM (2012). Novel nongenomic signaling by glucocorticoid may involve changes to liver membrane order in rainbow trout. PLoS One.

[25] Dindia L, Faught E, Leonenko Z, Thomas R, Vijayan MM (2013). Rapid cortisol signaling in response to acute stress involves changes in plasma membrane order in rainbow trout liver. Am. J. Physiol. Metab..

[26] Faught E, Henrickson L, Vijayan MM (2017). Plasma exosomes are enriched in Hsp70 and modulated by stress and cortisol in rainbow trout. J. Endocrinol..

[27] Strober W (2001). Trypan blue exclusion test of cell viability. Curr. Protoc. Immunol..

[28] Bootman MD, Rietdorf K, Collins T, Walker S, Sanderson M (2013). Ca^2+^-sensitive fluorescent dyes and intracellular Ca^2+^ imaging. Cold Spring Harb. Protoc..

[29] Minta A, Kao JP, Tsien RY (1989). Fluorescent indicators for cytosolic calcium based on rhodamine and fluorescein chromophores. J. Biol. Chem..

[30] Jamaluddin M, Banerjee PP, Manna PR, Bhattacharya S (1989). Requirement of extracellular calcium in fish pituitary gonadotropin release by gonadotropin hormone-releasing hormone. Gen. Comp. Endocrinol..

[31] Collatz MB, Rüdel R, Brinkmeier H (1997). Intracellular calcium chelator BAPTA protects cells against toxic calcium overload but also alters physiological calcium responses. Cell Calcium.

[32] Verbost PM, Flik G, Lock RA, Wendelaar Bonga SE (1987). Cadmium inhibition of Ca^2+^ uptake in rainbow trout gills. Am. J. Physiol. Integr. Comp. Physiol..

[33] Koyanagi S, Kusunose N, Taniguchi M, Akamine T, Kanado Y, Ozono Y, Masuda T, Kohro Y, Matsunaga N, Tsuda M (2016). Glucocorticoid regulation of ATP release from spinal astrocytes underlies diurnal exacerbation of neuropathic mechanical allodynia. Nat. Commun..

[34] Oka T, Sato K, Hori M, Ozaki H, Karaki H (2002). Xestospongin C, a novel blocker of IP3 receptor, attenuates the increase in cytosolic calcium level and degranulation that is induced by antigen in RBL-2H3 mast cells. Br. J. Pharmacol..

[35] O’Brien J, Valdivia HH, Block BA (1995). Physiological differences between the alpha and beta ryanodine receptors of fish skeletal muscle. Biophys.

[36] James RS, Little AG, Tallis J, Seebacher F (2016). Thyroid hormone influences muscle mechanics in carp (*Cyprinus carpio*) independently from SERCA activity. J. Exp. Biol..

[37] García-López A, de Jonge H, Nóbrega RH, de Waal PP, van Dijk W, Hemrika W, Taranger GL, Bogerd J, Schulz RW (2010). Studies in zebrafish reveal unusual cellular expression patterns of gonadotropin receptor messenger ribonucleic acids in the testis and unexpected functional differentiation of the gonadotropins. Endocrinology.

[38] Wellerdieck C, Oles M, Pott L, Korsching S, Gisselmann G, Hatt H (1997). Functional expression of odorant receptors of the zebrafish Danio rerio and of the nematode *C.**elegans* in HEK293 cells. Chem. Senses..

[39] Sawisky GR, Chang JP (2005). Intracellular calcium involvement in pituitary adenylate cyclase-activating polypeptide stimulation of growth hormone and gonadotrophin secretion in goldfish pituitary cells. J. Neuroendocrinol..

[40] Diercks B-P, Werner R, Weidemuller P, Czarniak F, Hernandez L, Lehmann C, Rosche A, Kruger A, Kaufmann U, Vaeth M, Failla AV, Zobiak B, Kandil FI, Schetelig D, Ruthenbeck A, Meier C, Lodygin D, Flugel A, Ren D, Wolf IMA, Feske S, Guse AH (2018). ORAI1, STIM1/2, and RYR1 shape subsecond Ca(2+) microdomains upon T cell activation. Sci. Signal..

[41] Eylenstein A, Gehring E-M, Heise N, Shumilina E, Schmidt S, Szteyn K, Munzer P, Nurbaeva MK, Eichenmuller M, Tyan L, Regel I, Foller M, Kuhl D, Soboloff J, Penner R, Lang F (2011). Stimulation of Ca^2+^-channel Orai1/STIM1 by serum- and glucocorticoid-inducible kinase 1 (SGK1). FASEB J..

[42] Zhang B, Yan J, Umbach AT, Fakhri H, Fajol A, Schmidt S, Salker MS, Chen H, Alexander D, Spichtig D, Daryadel A, Wagner CA, Foller M, Lang F (2016). NFkappaB-sensitive Orai1 expression in the regulation of FGF23 release. J. Mol. Med. (Berl.).

[43] Nesan D, Vijayan MM (2016). Maternal cortisol mediates hypothalamus-pituitary-interrenal axis development in zebrafish. Sci. Rep..

[44] Li Y, Liu Y, Liu B, Wang J, Wei S, Qi Z, Wang S, Fu W, Chen Y-G (2018). A growth factor-free culture system underscores the coordination between Wnt and BMP signaling in Lgr5(+) intestinal stem cell maintenance. Cell Discov..

[45] Chan CM, Aw JTM, Webb SE, Miller AL (2016). SOCE proteins, STIM1 and Orai1, are localized to the cleavage furrow during cytokinesis of the first and second cell division cycles in zebrafish embryos. Zygote.

[46] Nuccitelli R, Wilson L, Matsudaira PT (1994). A Practical Guide to the Study of Calcium in Living Cells.

[47] Thraya, M. Characterization of rapid nongenomic cortisol signalling in rainbow trout liver (2018).

[48] Solyom A, Lauter CJ, Trams EG (1972). Plasma membranes from isolated liver cells. Biochim. Biophys. Acta Biomembr..

[49] Gravel A, Wilson JM, Pedro DFN, Vijayan MM (2009). Non-steroidal anti-inflammatory drugs disturb the osmoregulatory, metabolic and cortisol responses associated with seawater exposure in rainbow trout. Comp. Biochem. Physiol. Part C Toxicol. Pharmacol..

[50] Bjørklund SS, Kristensen VN, Seiler M, Kumar S, Alnæs GIG, Ming Y, Kerrigan J, Naume B, Sachidanandam R, Bhanot G, Børresen-Dale A-L, Ganesan S (2015). Expression of an estrogen-regulated variant transcript of the peroxisomal branched chain fatty acid oxidase ACOX2 in breast carcinomas. BMC Cancer.

[51] Irwin JJ, Shoichet BK (2005). ZINC—a free database of commercially available compounds for virtual screening. J. Chem. Inf. Model..

[52] Faught E, Vijayan MM (2018). The mineralocorticoid receptor is essential for stress axis regulation in zebrafish larvae. Sci. Rep..

[53] Faught E, Best C, Vijayan MM (2016). Maternal stress-associated cortisol stimulation may protect embryos from cortisol excess in zebrafish. R. Soc. Open Sci..

[54] Tu MK, Levin JB, Hamilton AM, Borodinsky LN (2016). Calcium signaling in skeletal muscle development, maintenance and regeneration. Cell Calcium.

[55] Hyde GN, Seale AP, Grau EG, Borski RJ (2004). Cortisol rapidly suppresses intracellular calcium and voltage-gated calcium channel activity in prolactin cells of the tilapia (*Oreochromis mossambicus*). Am. J. Physiol. Endocrinol. Metab..

[56] Thévenod F (2018). Membrane transport proteins and receptors for cadmium and cadmium complexes. Cadmium Interaction with Animal Cells.

[57] Van Deurs B, Roepstorff K, Hommelgaard AM, Sandvig K (2003). Caveolae: Anchored, multifunctional platforms in the lipid ocean. Trends Cell Biol..

[58] Hou X, Pedi L, Diver MM, Long SB (2012). , Crystal structure of the calcium release–activated calcium channel Orai. Science.

[59] Valverde MA, Rojas P, Amigo J, Cosmelli D, Orio P, Bahamonde MI, Mann GE, Vergara C, Latorre R (1999). Acute activation of Maxi-K channels (hSlo) by estradiol binding to the β subunit. Science.

[60] King JT, Lovell PV, Rishniw M, Kotlikoff MI, Lou Zeeman M, McCobb DP (2006). β2 and β4 subunits of BK channels confer differential sensitivity to acute modulation by steroid hormones. J. Neurophysiol..

[61] Bukiya AN, Singh AK, Parrill AL, Dopico AM (2011). The steroid interaction site in transmembrane domain 2 of the large conductance, voltage- and calcium-gated potassium (BK) channel accessory β1 subunit. Proc. Natl. Acad. Sci. USA.

[62] Zhou Y, Meraner P, Kwon HT, Machnes D, Oh-Hora M, Zimmer J, Huang Y, Stura A, Rao A, Hogan PG (2010). STIM1 gates the store-operated calcium channel ORAI1 in vitro. Nat. Struct. Mol. Biol..

[63] Zhou Y, Cai X, Loktionova NA, Wang X, Nwokonko RM, Wang X, Wang Y, Rothberg BS, Trebak M, Gill DL (2016). The STIM1-binding site nexus remotely controls Orai1 channel gating. Nat. Commun..

[64] Zhou Y, Nwokonko RM, Baraniak JH, Trebak M, Lee KPK, Gill DL (2019). The remote allosteric control of Orai channel gating. PLoS Biol..

[65] Cain DW, Cidlowski JA (2017). Immune regulation by glucocorticoids. Nat. Rev. Immunol..

[66] Resende R, Fernandes T, Pereira AC, De Pascale J, Marques AP, Oliveira P, Morais S, Santos V, Madeira N, Pereira CF (2020). Mitochondria, endoplasmic reticulum and innate immune dysfunction in mood disorders: Do mitochondria-associated membranes (MAMs) play a role?. Biochim. Biophys. Acta Mol. Basis Dis..

