# Supplementary material for: Cortisol modulates calcium release-activated calcium channel gating in fish hepatocytes
Source: Sci Rep. 2021 May 5;11:9621. doi: 10.1038/s41598-021-88957-3 (PMC8100157; doi:10.1038/s41598-021-88957-3)
Supplement: Supplementary file 1 — Supplementary Information 1. [file 41598_2021_88957_MOESM1_ESM.docx]

**Supplementary Information**

**Cortisol Modulates Calcium Release-Activated Calcium**

**Channel Gating in Fish Hepatocytes**

Chinmayee Das, Manoj K. Rout, Willem C. Wildering and Mathilakath M. Vijayan*

**To whom correspondence should be addressed. Email:* [*matt.vijayan@ucalgary.ca*](mailto:matt.vijayan@ucalgary.ca)

**This file includes:**

Figs. S1-S4


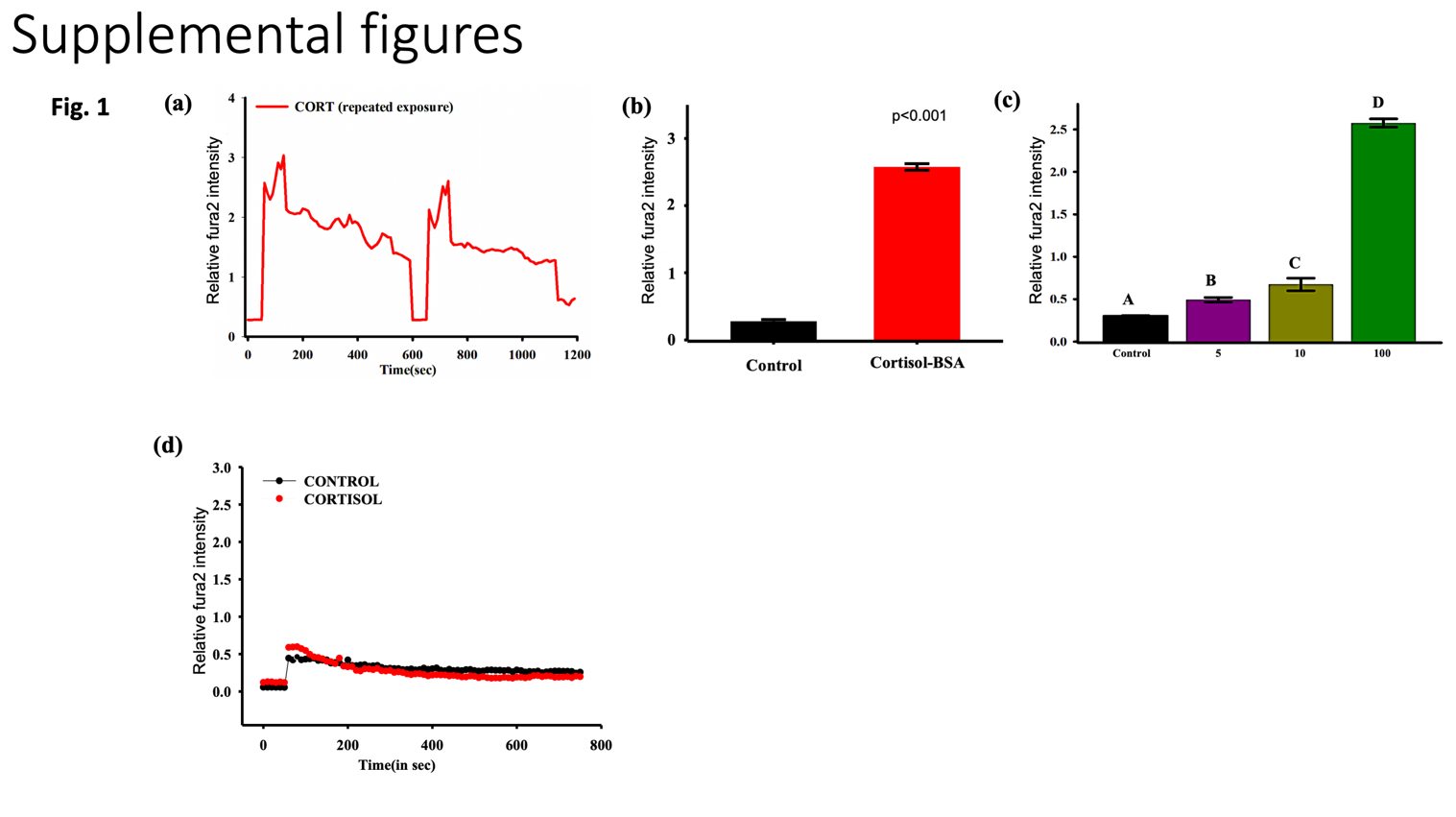


**Figure S1: Cortisol stimulation of Calcium wave: (a)** To determine cortisol action as transient and rapidly stimulated**,** repeated exposure to cortisol at a concentration of 100ng/ml was tested. Cortisol treated cells were washed thoroughly and were allowed to settle for 10min prior to second exposure. Before the exposure of cells to cortisol, cell calcium readings were taken to determine the basal levels of Ca^2+^. Repeated exposure resulted in a similar transient change to intracellular Ca^2+^ as seen initially. **(b)** Membrane impermeable form of cortisol was tested to determine cortisol action independent of any intracellular interaction. Bar graph showing ([Ca^2+^] i) changes at 60 s after cortisol-BSA addition (t-test; p<0.001). **(c)** CORT-BSA dosage study was conducted to determine concentration dependent or independent action. Bar graph showed a concentration dependent action of CORT-BSA**.** Dose-related ([Ca^2+^] i) response at 60 s to cortisol (0, 5,10 and 100ng/ml). Bar graph indicates CORT-BSA at a concentration of 100ng/ml showed a similar response as cortisol**. (d)** Without no extracellular calcium, control and cortisol showed a similar pattern of calcium alteration.


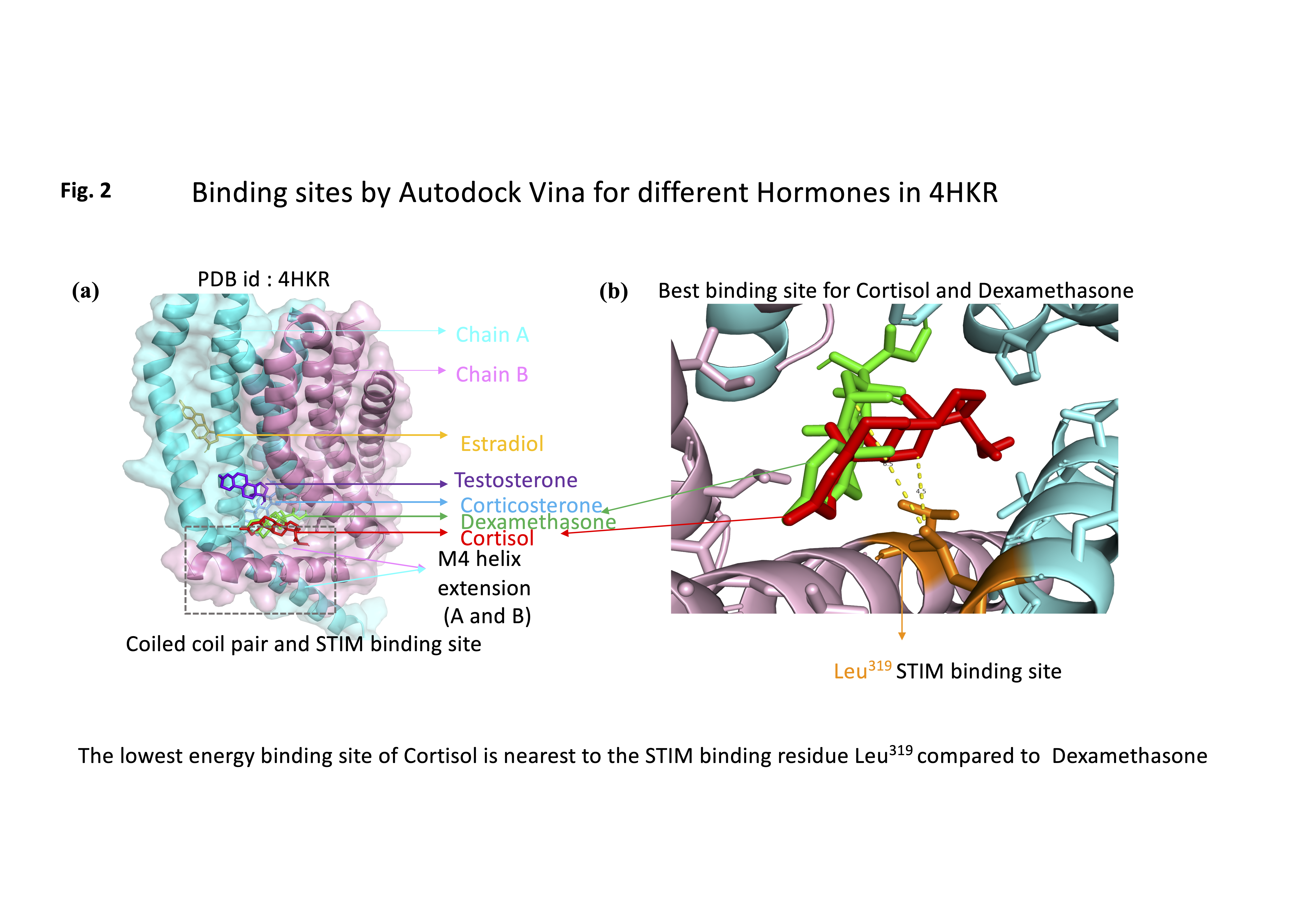


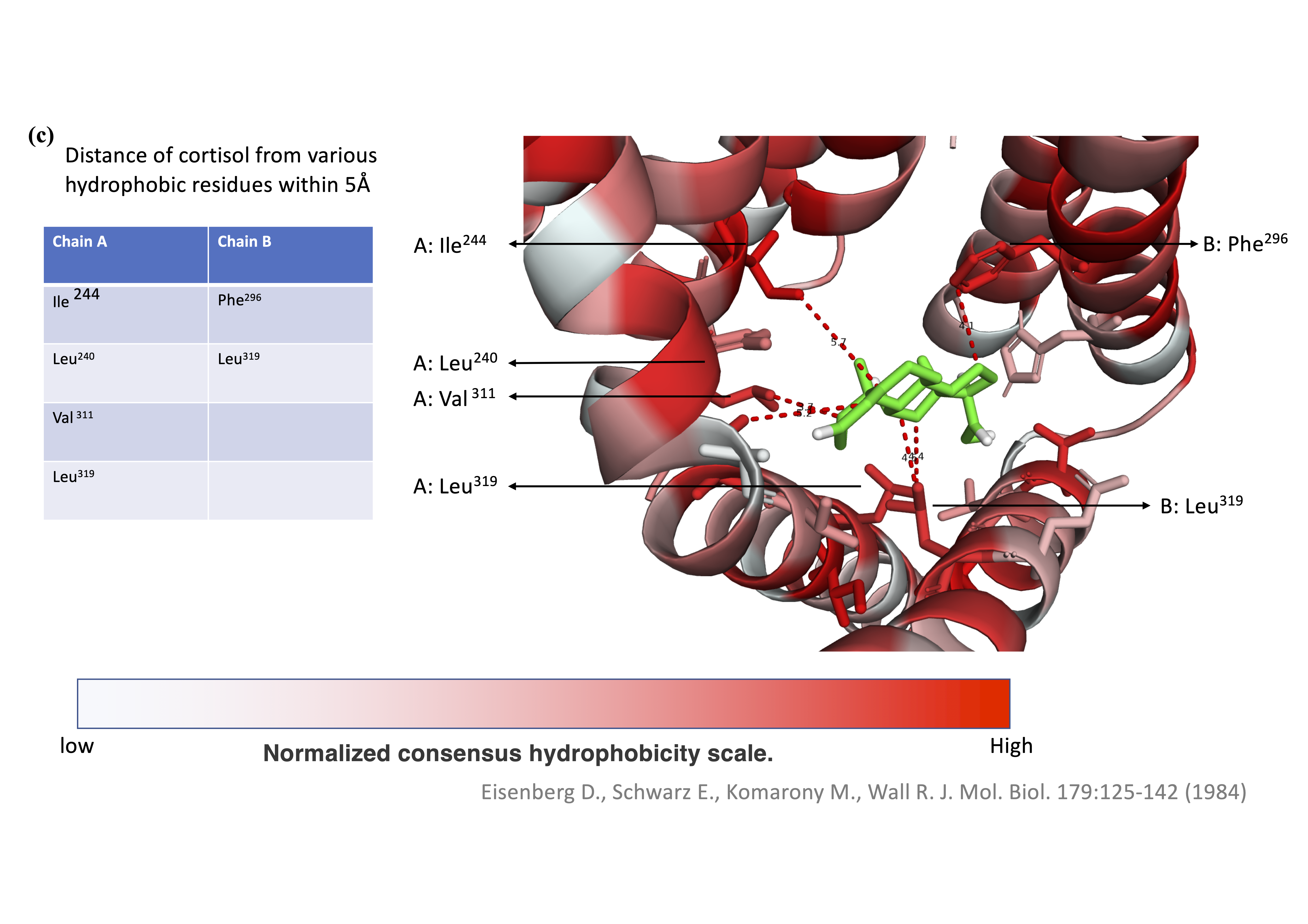


**Figure S2: Binding prediction sites by Autodock Vina for different Hormones in contrast to cortisol with CRAC channel protein.** To find the entire binding site on CRAC, the grid was modified to cover the entire protein (CRAC), as we are unaware of the binding sites. All the ligands selected for testing including cortisol, corticosterone, estradiol, testosterone and dexamethasone were downloaded from ZINC database (*49*). The *Drosophila melanogaster* CRAC channel (4HKR.pdb) was used as the receptor. CRAC channel structure is a hexameric assembly of four transmembrane helices (M1-M4) and helix extension of M4 extending into the cytosol. The channel pore is made up of six M1 helices to form the inner pore. M2 and M3 together form the outer lining for M1 helices and separate them from M4 helices. M4 helices are the peripheral outer ring subunit of CRAC that interacts with STIM for channel gating. Studies confirm that STIM binding to Leu^319^ or Ile^319^ at the M4 extension A and B is critical for channel activation. **(a)** Lowest energy binding sites of different hormones including testosterone (T), corticosterone (B), estradiol (E2), dexamethasone (DEX) with or without cortisol in CRAC predicted from docking calculation. The structure indicates the portion of CRAC interacting with hormones (cartoon representation). Out of all of them only cortisol and dexamethasone were near to the active site, which are the coiled-coiled pair and also the STIM binding site. **(b)** A more detail view of the binding confirmations of cortisol (red) and dexamethasone (green). Cortisol (distance ~ 4.6Å) is closer to the Leu^319^ (orange) of the STIM binding site than dexamethasone (distance ~ 6.5Å) predicting a higher probability of interaction of cortisol with the binding site. **(c)** Intermolecular interactions play a critical role in ligand receptor interaction from an energetically stabilizing perspective (*50*). Increased interaction of cortisol with hydrophobic residues indicates a much stable prediction for binding at the ORAI channel-gating site at the atomic level. Distance of cortisol from various hydrophobic residues within 4.5Å. The cortisol binding site residues of CRAC are coloured from low (white) to high (red) based on normalized consensus hydrophobicity scale. (Left) various hydrophobic residues from chain A and B are shown in table. (Right) these residues are represented by sticks along with their position in CRAC. The corresponding distances from cortisol (colored in green) showing the interacting hydrophobic chain are shown in dotted lines (red).


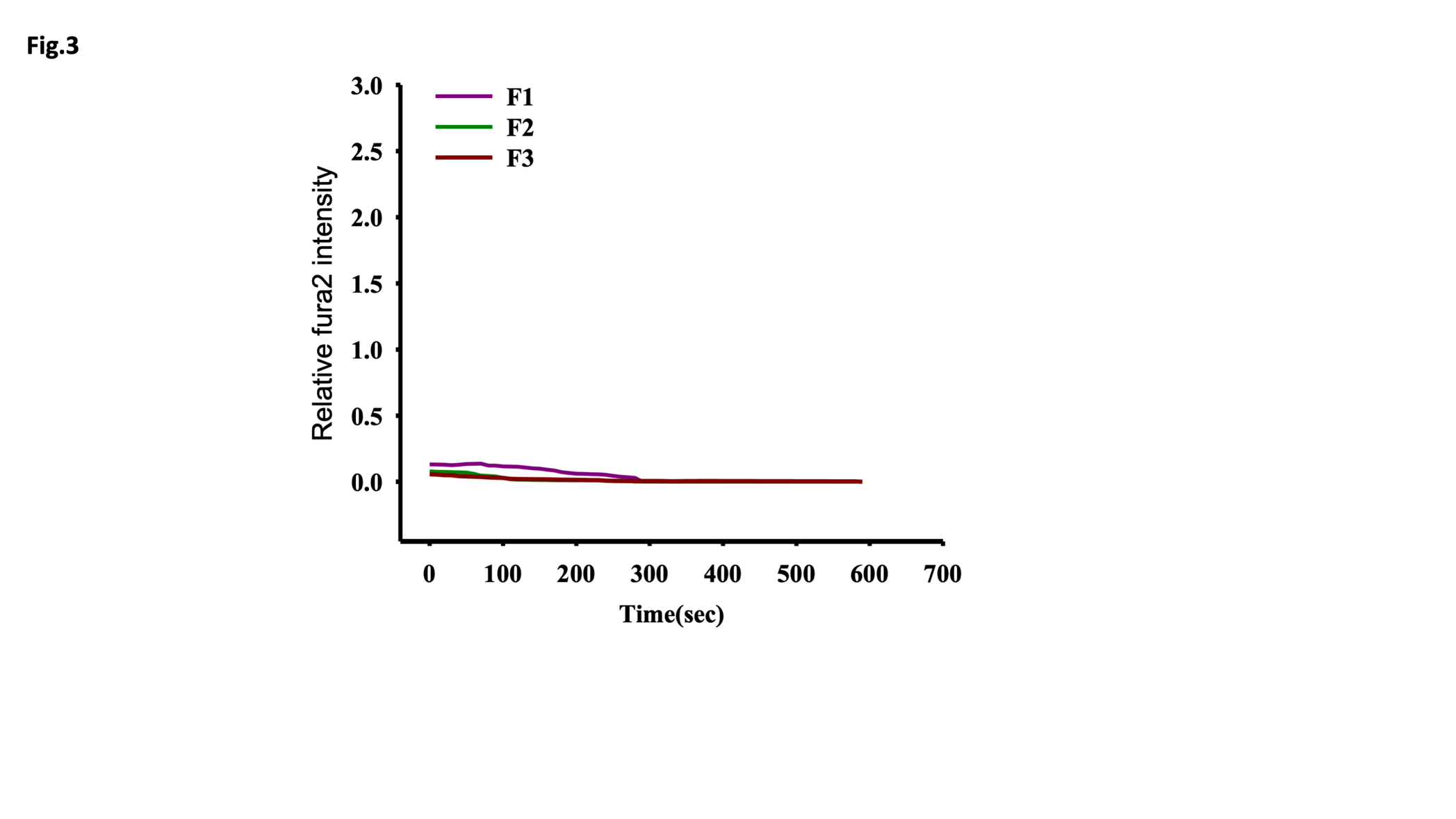


**Figure S3: Dye compartmentalization.** To assess whether there were any FURA 2-AM compartmentalization in the cellular organelles, Triton-100 was used. Permeabilization of cells with Triton showed no entrapment of FURA indicator inside the cells, indicating the cellular system to be an ideal model for investigation. The graph represents cells incubated with tritonx100 from three individual fish (F1-3) tested for compartmentalization.

^
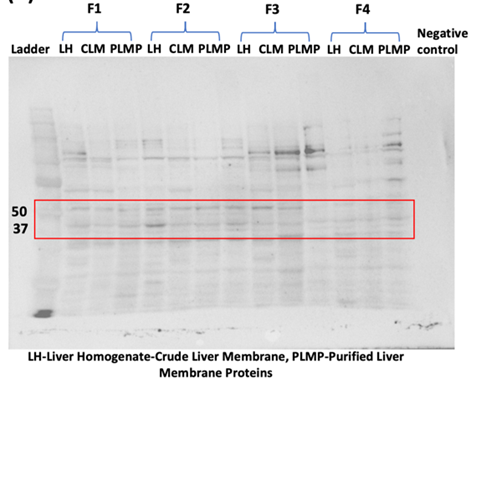
^

**Figure S4: ORAI1 protein expression in liver preparations. Dye compartmentalization.** A representative blot showing ORAI1expression (~51 Kda) in liver homogenate (LH), crude liver membranes (CLM) and purified liver membrane proteins (PLMP) from rainbow trout using immunodetection.
